# Accelerated magnetic resonance fingerprinting using soft-weighted key-hole (MRF-SOHO)

**DOI:** 10.1371/journal.pone.0201808

**Published:** 2018-08-09

**Authors:** Gastao Cruz, Torben Schneider, Tom Bruijnen, Andreia S. Gaspar, René M. Botnar, Claudia Prieto

**Affiliations:** 1 School of Biomedical Engineering and Imaging Sciences, King’s College London, London, United Kingdom; 2 Philips Healthcare, Guilford, United Kingdom; 3 Department of Radiotherapy, University Medical Center Utrecht, Utrecht, The Netherlands; 4 Institute for Systems and Robotics / Department of Bioengineering, Instituto Superior Técnico, Universidade de Lisboa, Lisbon, Portugal; 5 Escuela de Ingeniería, Pontificia Universidad Católica de Chile, Santiago, Chile; Johns Hopkins School of Medicine, UNITED STATES

## Abstract

**Object:**

To develop a novel approach for highly accelerated Magnetic Resonance Fingerprinting (MRF) acquisition.

**Materials and methods:**

The proposed method combines parallel imaging, soft-gating and key-hole approaches to highly accelerate MRF acquisition. Slowly varying flip angles (FA), commonly used during MRF acquisition, lead to a smooth change in the signal contrast of consecutive time-point images. This assumption enables sharing of high frequency data between different time-points, similar to what is done in some dynamic MR imaging methods such as key-hole. The proposed approach exploits this information using a SOft-weighted key-HOle (MRF-SOHO) reconstruction to achieve high acceleration factors and/or increased resolution without compromising image quality or increasing scan time. MRF-SOHO was validated on a standard T_1_/T_2_ phantom and in *in-vivo* brain acquisitions reconstructing T_1_, T_2_ and proton density parametric maps.

**Results:**

Accelerated MRF-SOHO using less data per time-point and less time-point images enabled a considerable reduction in scan time (up to 4.6x), while obtaining similar T_1_ and T_2_ accuracy and precision when compared to zero-filled MRF reconstruction. For the same number of spokes and time-points, the proposed method yielded an enhanced performance in quantifying parameters than the zero-filled MRF reconstruction, which was verified with 2, 1 and 0.7 (sub-millimetre) resolutions.

**Conclusion:**

The proposed MRF-SOHO enabled a 4.6x scan time reduction for an in-plane spatial resolution of 2x2 mm^2^ when compared to zero-filled MRF and enabled sub-millimetric (0.7x0.7 mm^2^) resolution MRF.

## Introduction

Quantitative Magnetic Resonance Imaging (MRI) techniques, such as T_1_, T_2_ and proton density (M_0_) parametric maps, have been developed to enable direct objective comparison and characterization of diseased and healthy tissue [1–6]. However, current quantitative MRI methods are slow and typically provide single parameter information per acquisition. Therefore different parameters need to be measured in serial acquisitions, further increasing the scan time. Magnetic Resonance Fingerprinting (MRF) [7] has been recently introduced to retrieve multiple and simultaneous parametric maps from a single acquisition under the assumption that unique signal evolutions or “fingerprints” can be generated from different tissues. This is achieved using a variable encoding and acquisition scheme that typically varies flip angles (FA) and repetition times (TR) throughout the scan. Since there are little constraints regarding the acquired signal, MRF is exceptionally flexible in terms of pulse sequence design. Multiple parameters are reconstructed in MRF via pixel-wise template matching of a measured signal (series of time-point images) to a previously generated dictionary of signals. Generally, template matching is achieved by finding the dictionary entry with the highest inner product with the fingerprint in a given pixel, via exhaustive search. The dictionary needs to contain signal evolutions from all potential tissues of interest subject to a set of sequence parameters, thus all MR parameters employed to generate the dictionary of fingerprints can be reconstructed simultaneously. Dictionaries have been generated using Bloch equation or extended phase graph formalisms to simulate the acquisition sequence [8]. MRF reconstruction has been shown to be robust to measurement noise and incoherent undersampling artefacts, provided a large number (~1000) of time-point images are acquired, and therefore highly undersampled time-point images have been employed [7]. In spite of the high undersampling factors reported, the large amount of time point images required in MRF may result in long acquisitions for some important applications such as high-resolution 2D breath hold acquisitions and high-resolution 3D scans. Moreover the low quality of the highly undersampled time-point images can introduce bias in the parametric values if the template matching based MRF reconstruction fails to distinguish between signal and noise or undersampling artefacts [9].

Recent work has focused on investigating the feasibility of MRF for different applications, as well as improving the MRF framework itself. MRF has been combined with simultaneous multi-slice acquisition [10] at an acceleration factor of two and with echo-planar imaging [11] to simultaneously measure T_1_ and T_2_*. B_1_ estimation has been incorporated into the signal simulation in 2D and 3D MRF, enabling measurements of T_1_, T_2_ and B_1_ with increased accuracy [12, 13]. Myocardial MRF in 2D using magnetization preparation pulses and cardiac triggering has been developed, showing good agreement with gold standard methods [14]. Template matching durations have also been significantly reduced using fast group matching algorithms [15]. In [16], a multi-scale MRF reconstruction was introduced, improving measurement accuracy and significantly reducing the number of time-point images required. More recently a sliding window reconstruction was proposed to improve T_1_ and T_2_ measurement accuracy and/or reduce acquisition time [17]. The low rank nature of the dictionary has been leveraged to improve parametric map quality and reduce the number of Fourier transforms required [18]. A maximum likelihood formulation has been introduced [19], also accelerating the number of time-points. With the high acceleration factors typically employed in MRF (R>20), the parallel imaging reconstruction is very ill-posed. With sufficient a priori information the linear problem can be solved, producing high quality time-point images and consequently improving the parametric maps.

In this work, we develop a novel reconstruction approach for highly accelerated MRF acquisition. The proposed SOft-weighted key-HOle (MRF-SOHO) reconstruction enables shorter acquisition times (higher acceleration factor per time-point image and/or reduced number of time-points), and improved sub-millimetre in-plane resolution. This is achieved by considerably improving the quality of the time-point images with a reconstruction based on parallel imaging [20], soft-gating [21] and key-hole [22] methods. Slow FA variation between time-point images is commonly used in MRF to explore the parametric space, generating unique fingerprints. This continuous change in magnetization leads to a smooth change in contrast through the time-point images, resulting in redundant high spatial frequency information. MRF-SOHO exploits this redundancy by using a soft-weighted iterative SENSE reconstruction to share high-frequency data between consecutive time-point images. The proposed approach was validated on a standardized T_1_/T_2_ phantom and in *in-vivo* brain data in three healthy subjects.

## Materials and methods

### Data acquisition

Gradient echo readout with variable FA and TR after an initial inversion pulse was used for data acquisition as proposed in [23]. A golden radial trajectory [24] was employed instead of the spiral trajectory used in [23]. However, multiple radial spokes may be required per time-point image. To this end, the acquisition scheme in [23] was modified such that the FA and TR corresponding to time point *t* (FA_t_ and TR_t_, respectively) were repeated N_p_ times, where N_p_ is the number of radial spokes per time-point image. The FA was given by:

$${FA}_{t}=sin\left(\frac{\lceil t/N_{p}+1\rceil \pi }{N_{rf}}\right){FA}_{max}$$

where N_rf_ is the number of RF pulses per section of the acquisition and FA_max_ is the maximum FA in the corresponding section. The TR pattern followed a pseudo random Perlin noise. Both FA and TR patterns are further described in [23].

### Reconstruction of time-point images

**Fig 1:**
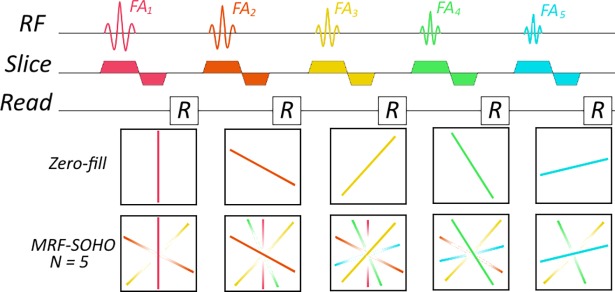
Pictorial diagram of MRF-SOHO’s data sharing scheme. The example depicted features a neighbourhood size (N) of 5, with one radial profile per flip angle (N_p_). The transparency of colour indicates the fraction of data shared. The farther away the time-point, the less low frequency data is shared.

**Fig 2:**
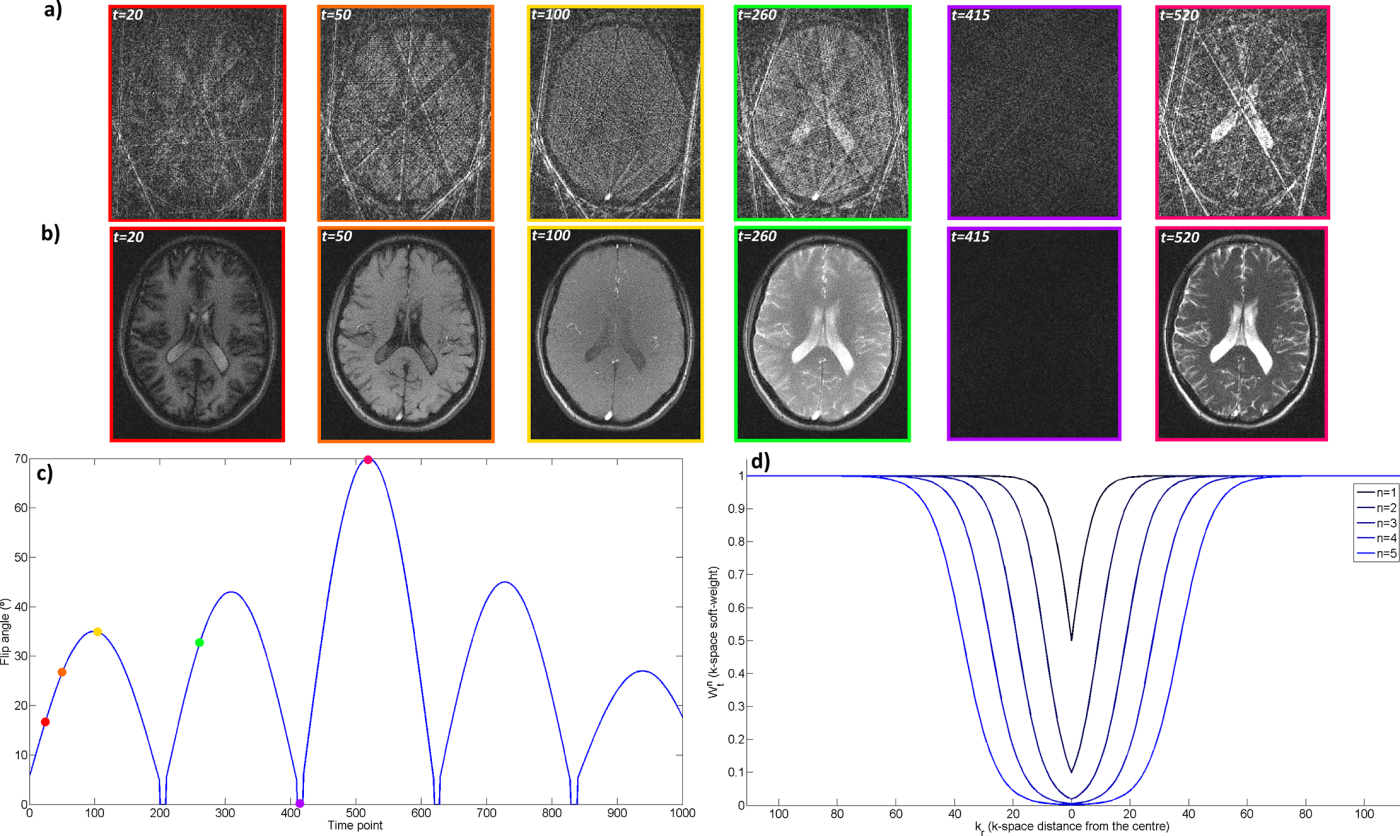
Time-points, flip angle pattern and soft-weights. **a)** Gridding reconstruction used in zero-filled MRF at different time-points in the acquisition, with 8 spokes per time-point. **b)** MRF-SOHO reconstruction at different time-points in the acquisition. High quality images are reconstructed at each time point with MRF-SOHO without affecting image contrast. **c)** Flip angle pattern used presents a smooth evolution. Coloured time-points correspond to the images above. **d)** Soft-weights ($W_{t}^{n}$) for different neighbouring time-points (*n*) as a function of k-space radial distance (*k*_*r*_). The further away the time point, the less low frequency data is shared.

The proposed MRF-SOHO reconstruction relies on high-frequency data redundancy of neighbouring time-point images under the assumption of smoothly varying contrast. Information is shared between neighbouring time-point images ***I***_*t*_ using an iterative SENSE soft-weighted reconstruction given by:

$${\hat{I}}_{t}={arg\;min}_{I_{t}}\{{\left\|W_{t}^{n}(FSI_{t}-K)\right\|}_{2}^{2}\}$$

where ***F*** is the Fourier transform (including non-Cartesian density compensation and non-uniform Fourier transform), ***S*** are the coil sensitivities and ***K*** are the acquired k-space data for all time-points. Equation 2 was solved with the Conjugate Gradient method. The soft-weights $W_{t}^{n}$ for time-point *t* and neighbor *n* ∈ {*t* − Δ*t*,*t* − Δ*t* + 1,…,*t* + Δ*t* −1,*t* + Δ*t*} are given by:

$$W_{t}^{n}(k_{r})=G_{t}^{n}∙\left\{1-{\left[\exp\left(\frac{k_{r}-\alpha (n)}{\beta (n)}\right)+1\right]}^{-1}\right\},\;if\;n\neq t$$

$$W_{t}^{n}(k_{r})=1\;,\;if\;n=t$$

$$G_{t}^{n}={\left[\exp\left(\frac{\frac{\left|{FA}_{t}-{FA}_{n}\right|}{{FA}_{t}}-\mu }{\tau }\right)+1\right]}^{-1}$$

where *k*_*r*_ is the k-space distance from the corresponding sample to the k-space centre, *α* is the distance at which $W_{t}^{n}(\alpha )=0.5$ and *β* determines the smoothness of the transition from $W_{t}^{n}(k_{r})≅0$ to $W_{t}^{n}(k_{r})≅1.\;G_{t}^{n}$ determines how much data is shared as a function of the relative change in the FA: the faster the rate of change in FA, the faster the change in contrast and therefore the less the data that can be shared. *μ* determines the threshold where data sharing is reduced and *τ* determines the smoothness of that transition. Through parameters *α*, *β*, *μ*, *τ* and Δ*t* this distribution can be adjusted to adequately share data between neighbouring time-points without compromising image contrast. A pictorial diagram of the data shared can be seen in Fig 1 with one radial spoke per excitation and a neighbourhood size (*N* = 2Δ*t* + 1) of 5. Some high spatial frequency data is always shared within the neighbourhood, however the amount of low frequency data shared depends on the distance between time-point neighbours. Soft-weights, FAs and corresponding time-point images are shown in Fig 2. Representative time-point reconstructions with gridding, as in zero-filled MRF, and with MRF-SOHO demonstrate an improvement in time-points image quality while maintaining contrast. An example plot of soft-weights for N = 11 is shown in Fig 2, indicating the fraction of data shared as a function of the k-space distance to the centre. A plot of the flip angle pattern used and an example plot of the soft-weighting functions are also shown in Fig 2.

### Dictionary and pattern recognition

The MRF dictionaries were simulated using the Extended Phase Graph (EPG) formalism [8, 25], based on code available in [25] for different T_1_ and T_2_ values. B_1_ and RF slice profile were not incorporated into the dictionary. T_1_, T_2_ and M_0_ parametric maps were obtained via template matching as in [23]. The inner product between the temporal signal from each pixel and all dictionary entries was performed. The highest inner product indicated the correct dictionary entry for each pixel. M_0_ parametric maps were calculated as the ratio between the signal and the normalized dictionary entry for each pixel.

## Experiments

### Data acquisition

Phantom and *in-vivo* brain data was acquired on a 1.5T Ingenia MR system (Philips, Best, The Netherlands) using a 12-element head coil. The study was approved by the institutional review board (London Bridge Research Ethics Committee) and written informed consent was obtained from all subjects according to institutional guidelines.

2D acquisitions were performed on a standardized T_1_/T_2_ phantom [26] and in three healthy subjects. For both phantom and *in-vivo* acquisitions data was acquired with a gradient echo readout after an initial inversion pulse using a golden radial trajectory. In each case acquisitions were performed with three different in-plane resolutions of 1) 2x2 mm^2^, 2) 1x1 mm^2^ and 3) 0.7x0.7 mm^2^. The respective TRs varied between 1) 3.2 and 4.0 ms, 2) 4.7 and 5.7 ms, and 3) 5.8 and 7.2 ms. The respective TEs were: 1) 1.31 ms, 2) 1.85 ms and 3) 3.2 ms. The remaining relevant parameters were the same for all three acquisitions: 10 mm slice thickness, 260x260 mm^2^ field-of-view, 8 radial spokes per time-point, 1000 time points, one slice. The FA pattern was divided into sections of 200 time-points, with corresponding FA_max_ of 35, 43, 70, 45 and 27 degrees.

### Reconstruction of time-point images

Data from the phantom and one subject were reconstructed with different neighbourhood sizes (*N*), *α*(*n*), *β*(*n*), *μ* and *τ* parameters in order to experimentally tune these values. The reconstructions were visually inspected to select the appropriate parameters. Consequently, the MRF-SOHO reconstruction neighbourhood size (*N* = 2Δ*t* + 1) was set to 31, *α*(*n*) varied linearly between 0 and 20% *K*_*max*_, *β*(*n*) varied linearly between 1% *K*_*max*_ and 2% *K*_*max*_, where *K*_*max*_ is the index of the maximum k-space radial point. *μ* and *τ* were set to 0.2 and 0.05, respectively. Density compensation function for each time point was obtained via Voronoi diagrams [27] and non-uniform fast Fourier transform based on [28] was employed. The reconstruction was terminated when the relative residual reached 0.05% or when 15 iterations were reached (whichever came first). Coil maps were obtained from all the acquired data using ESPIRiT [29]. The acquired datasets were also reconstructed with a zero-filled gridding reconstruction as in [23]. Both MRF-SOHO and zero-filled MRF reconstructions were performed using 8, 5 and 2 radial profiles per time-point (retrospectively undersampled), corresponding to highly undersampled data. For instance, the 2x2 mm^2^ datasets with 8, 5 and 2 radial profiles per time-point have angular undersampling factors of ~26x, ~41x and ~102x (with respect to a fully sampled radial acquisition) respectively, whereas the 0.7x0.7 mm^2^ datasets with 8, 5 and 2 radial profiles per time-point have angular undersampling factors of ~73x, ~117x and ~292x (with respect to a fully sampled radial acquisition) respectively. Reconstructions were performed offline in MATLAB (Mathworks, Natick, Massachusetts, USA). MRF-SOHO reconstruction took approximately 10, 30 and 120 minutes to complete for the 2x2, 1x1 and 0.7x0.7 mm^2^ resolution datasets using 8 radial profiles and 1000 time points, respectively. These reconstructions ran on a Linux workstation with 12 Intel Xeon X5675 (3.07 GHz) and 200 GB RAM.

### Dictionary and pattern recognition

Dictionaries were tailored for the T_1_/T_2_ phantom and the brain datasets based on the corresponding tissues. For the phantom and brain data, T_1_ ∈ [0:10:400 ms, 400:5:800 ms, 800:20:1400 ms, 1400:200:6000 ms], T_2_ ∈ [0:1:150 ms, 150:10:500 ms, 500:50:1000 ms, 1000:200:2600 ms], corresponding to 35496 dictionary entries. For both simulations, B_1_ was fixed to 1.0. Template matching of the reconstructed datasets was performed using a different number of time-point images ranging from 50 to 1000 images (with a step size of 50) for both MRF-SOHO and zero-filled MRF.

## Results

A considerable improvement in image quality of the time-point images was observed with MRF-SOHO when compared to the zero-filled gridding reconstruction (zero-filled MRF), which is strongly dominated by undersampling artefacts. This can be seen in S1, S2 and S3 Videos, showing the first 500 time-point images reconstructed with zero-filled MRF and MRF-SOHO (using 8 spokes per time-point) in 2 mm^2^, 1 mm^2^ and 0.7 mm^2^, respectively, for subject 1. Inspecting the time-points corresponding to the lowest FA confirms that image contrast is generally unaffected with the proposed approach. Additionally, a variation in image quality can be observed depending on the amount of data shared: regions where contrast changes rapidly (e.g. when the flip angle goes to zero) share less data and therefore have increased residual aliasing.

**Fig 3:**
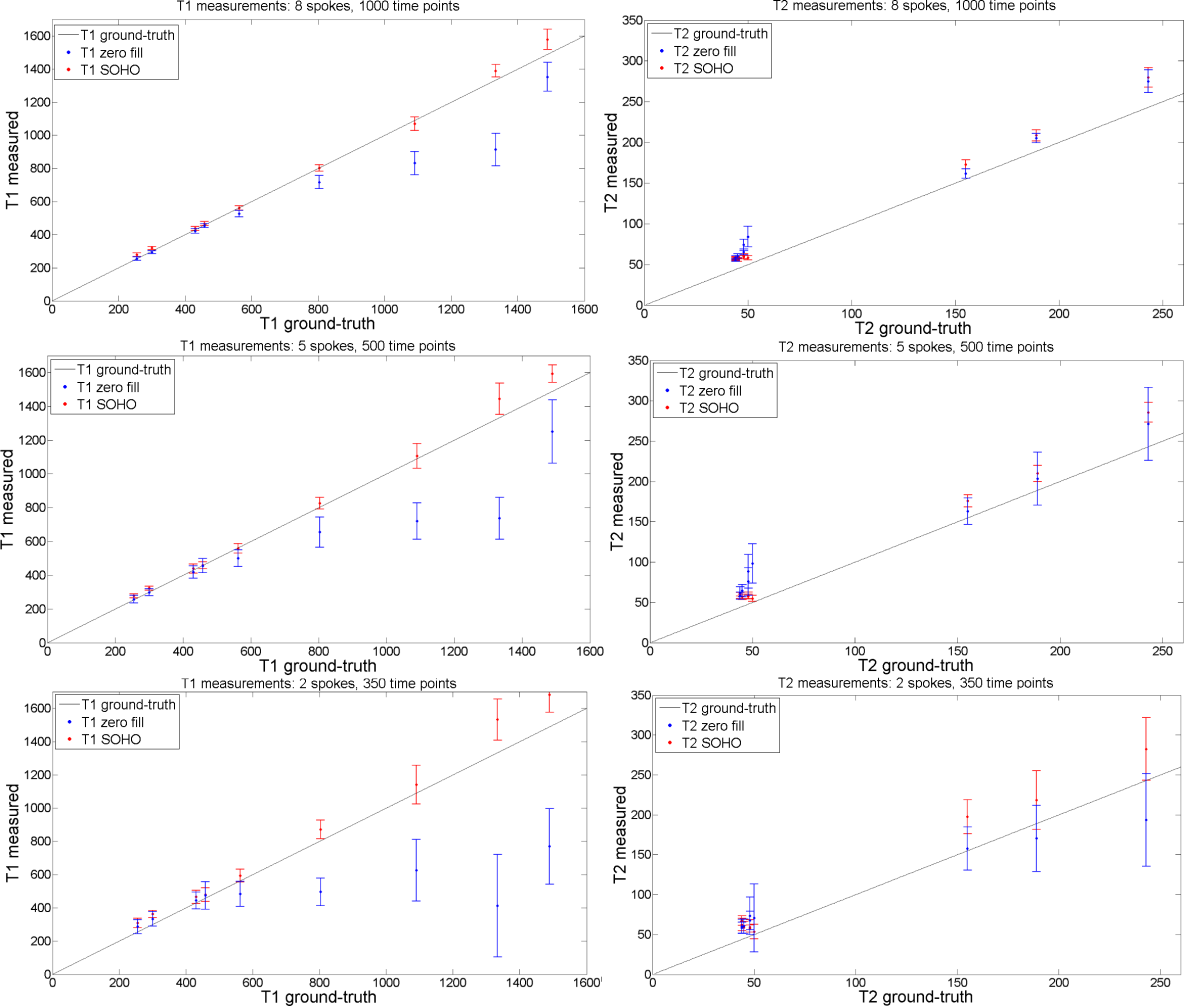
T_1_ and T_2_ measurement plots (in ms) with zero-filled MRF and the proposed MRF-SOHO reconstructions, using 8 spokes, 1000 time points; 5 spokes, 500 time points; and 2 spokes, 350 time points. An underestimation of T_1_ was observed for zero-filled MRF and an overestimation of T_2_ was observed for both methods. MRF-SOHO produced more accurate and precise measurements than zero-filled MRF, particularly when less data (time points and radial spokes) was used. Ground truth values were used according to (26).

T_1_, T_2_ parametric maps were reconstructed with zero-filled MRF and the proposed MRF-SOHO in the standardized phantom and compared with ground truth values. Plots of the measured T_1_ and T_2_ values in the phantom 2x2 mm^2^ dataset for reconstructions with different amounts of time-point images and with different undersampling factors per time-point image are shown in Fig 3. MRF-SOHO achieved superior accuracy and precision compared to zero-filled MRF for both T_1_ (particularly at high T_1_ values) and T_2_ (particularly at low T_2_ values). MRF-SOHO proved robust to higher acceleration factors, whereas zero-filled MRF lost precision and accuracy (particularly underestimation) at higher acceleration factors. Overall, a T_2_ overestimation was observed for both methods. A visual inspection (not shown) of the parametric maps with different accelerations revealed MRF-SOHO can be accelerated to 350 time-points using 5 radial spokes while maintaining parametric quality, in line with observations in vivo as shown below.

**Fig 4:**
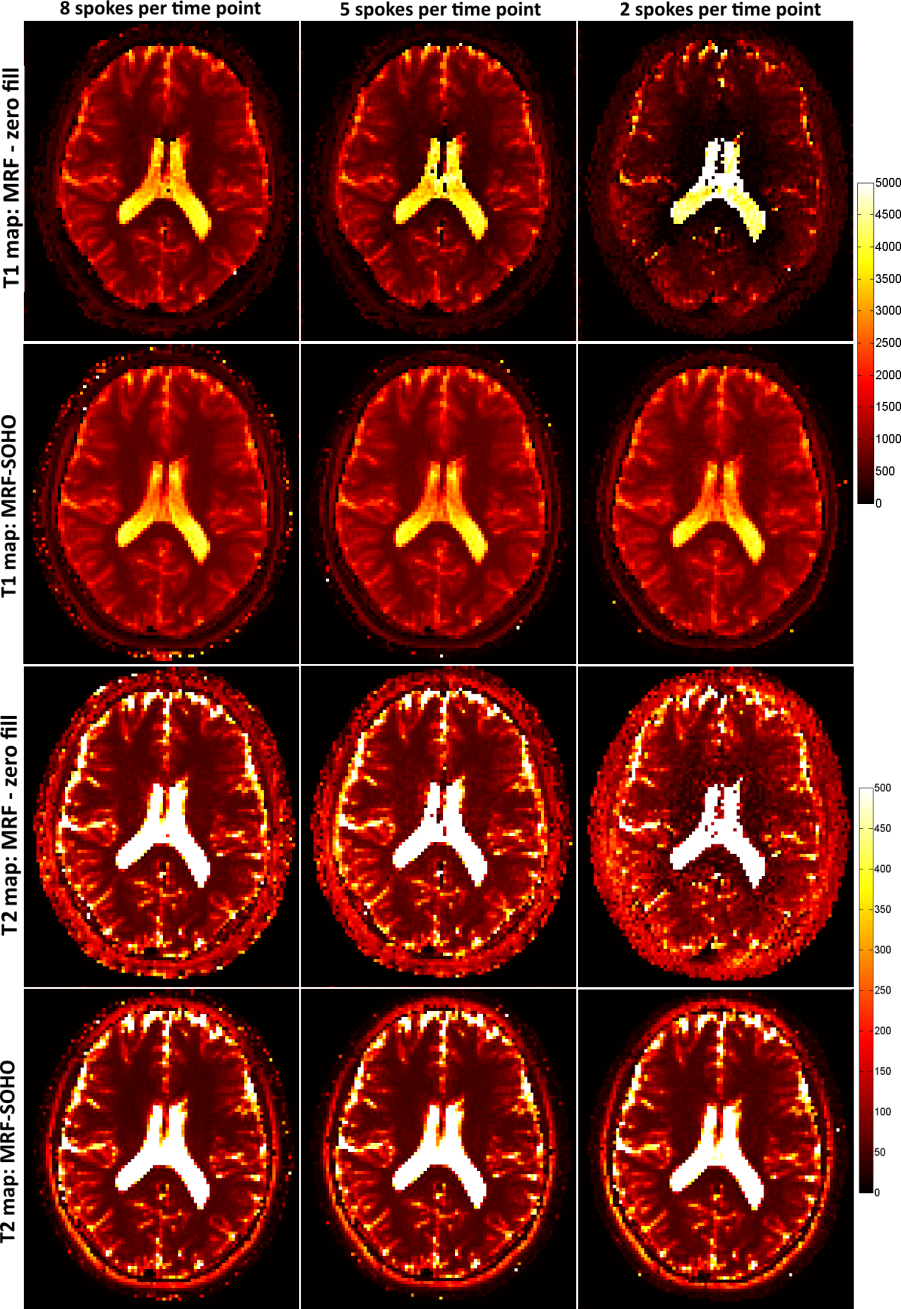
T_1_ and T_2_ maps (in ms) for zero-filled MRF reconstruction and the proposed MRF-SOHO reconstruction using 1000 time points and different number of radial spokes (8, 5 and 2) for subject 1. The zero-filled MRF reconstruction produces good quality parametric maps with 8 radial spokes, but quickly deteriorates with increased undersampling factor per time-point image. The proposed MRF-SOHO reconstruction produces good quality parametric maps in all cases, however the signal-to-noise ratio is decreased for reduced number of spokes per time point.

**Fig 5:**
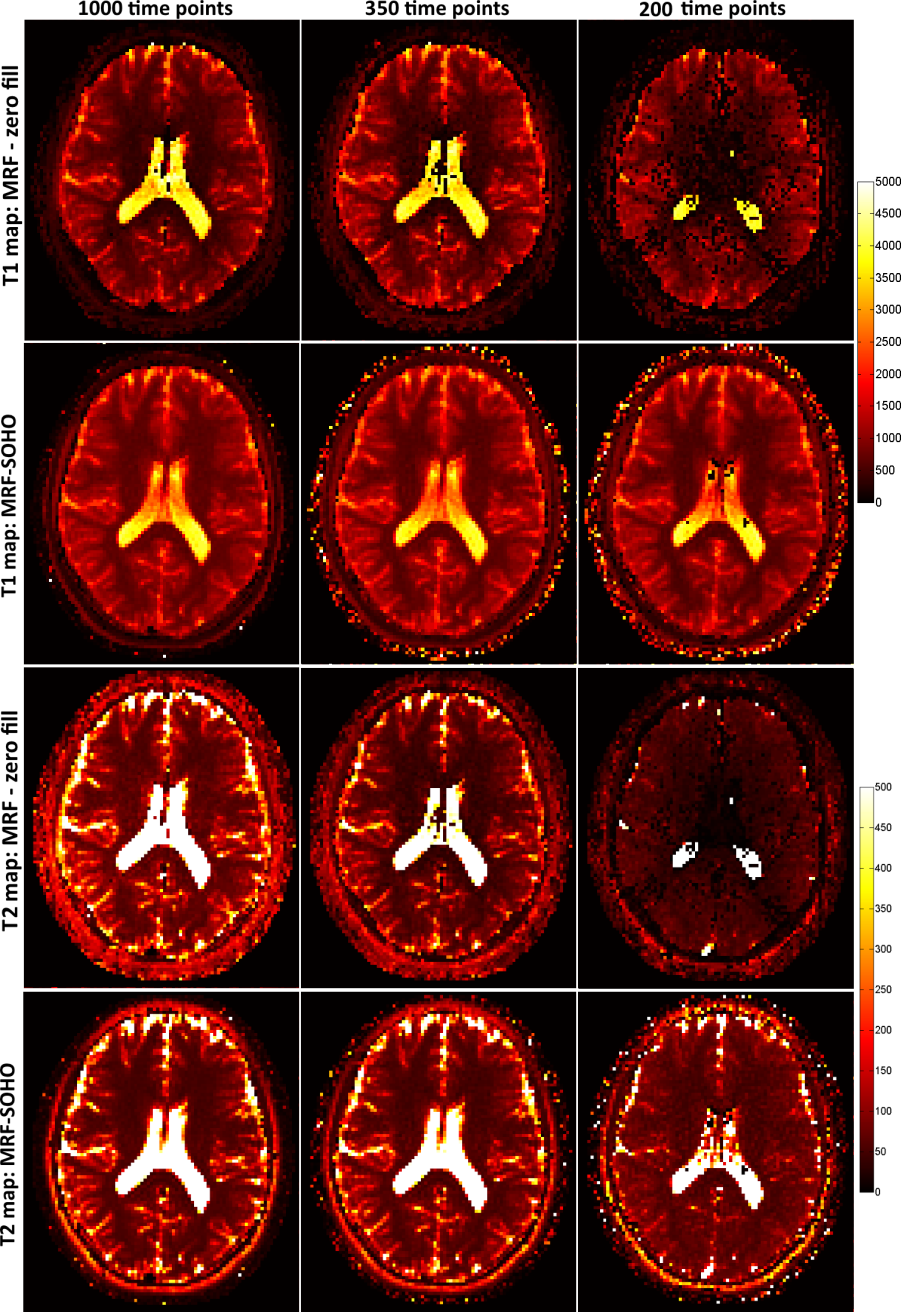
T_1_ and T_2_ maps (in ms) for zero-filled MRF reconstruction and the proposed MRF-SOHO reconstruction using 5 radial spokes and different number of time points (1000, 350 and 200). The zero-filled MRF reconstruction produces good quality parametric maps with 1000 time points, but starts failing with reduced number of time-point images. The proposed MRF-SOHO reconstruction produces good quality and accurate parametric maps in all cases, however the signal-to-noise ratio is reduced for decreased numbers of time points.

T_1_ and T_2_ maps for 2x2 mm^2^ dataset using 8, 5 and 2 radial profiles per time-point image and 1000 time-points are shown in Fig 4 for subject 1. Zero-filled MRF achieves good quality T_1_ and T_2_ maps with 8 radial profiles, but starts failing with higher undersampling factors (5 and 2 radial profiles per time-point image). Conversely, MRF-SOHO achieved superior quality parametric mapping across all undersampling factors, although a slight noise amplification can be observed at higher acceleration factors. Corresponding parametric maps for the same dataset using 1000, 350 and 200 time-point images with 5 radial profiles per time-point are shown in Fig 5. Analogous to the previous figure, the performance of zero-filled MRF breaks down with a reduced number of time-point images. MRF-SOHO proved more robust, however a loss in parametric map quality was observed with 200 time points. These images indicate parametric map quality decreases considerably when less than 350 time-points and less than 5 radial profiles are used, which is in agreement with the phantom data.

**Fig 6:**
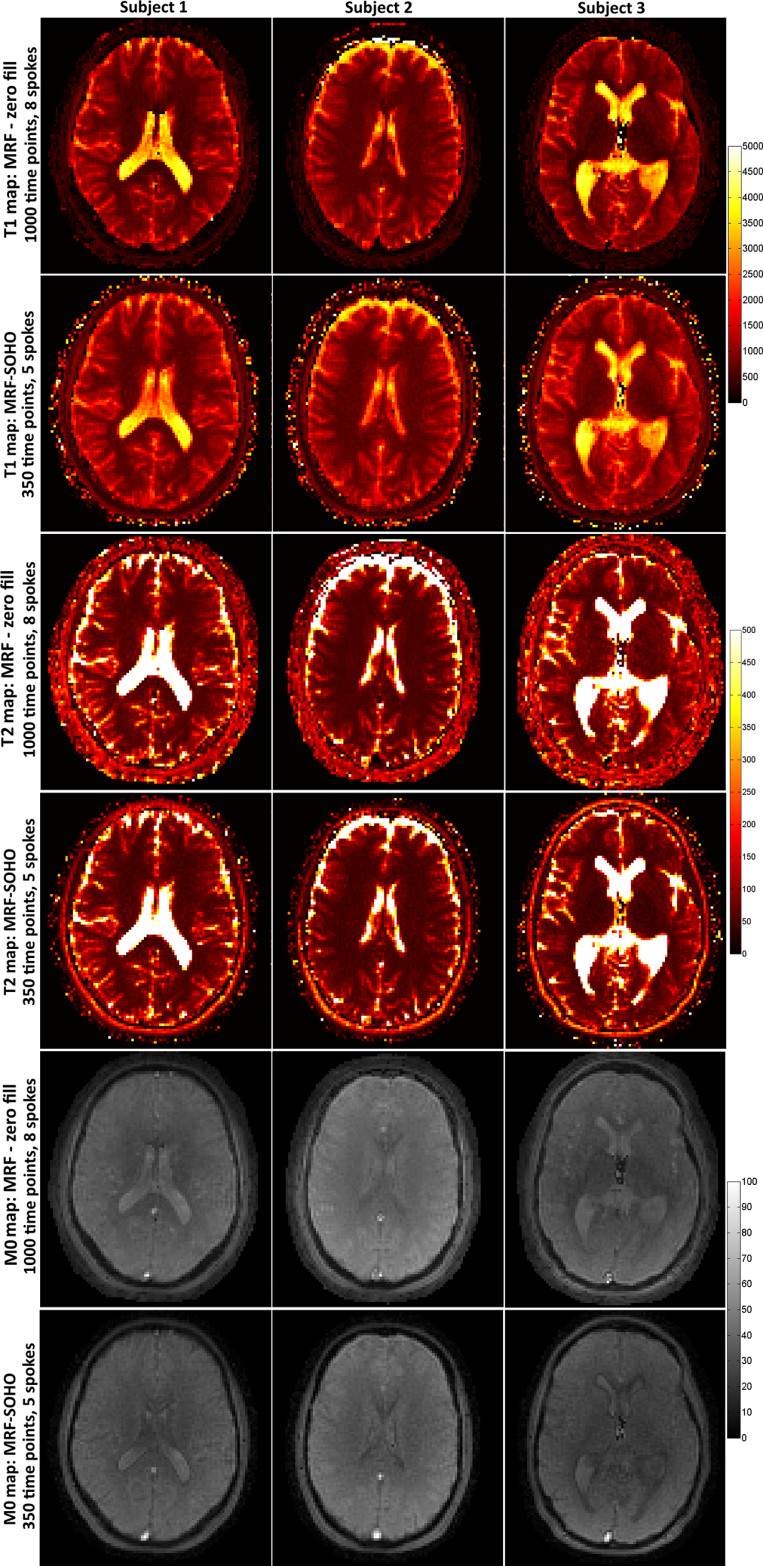
T_1_, T_2_ (in ms) and M_0_ maps for zero-filled MRF reconstruction (8 spokes, 1000 time-points) and the proposed accelerated MRF-SOHO (5 spokes, 350 time points). The accelerated MRF-SOHO produces similar parametric maps despite a ~4.6x relative acceleration factor to zero-filled MRF.

Accelerated MRF-SOHO T_1_, T_2_ and M_0_ parametric maps using 5 radial profiles and 350 time-points are shown in Fig 6 in comparison to the best zero-filled MRF reconstruction (i.e. 8 radial profiles per time-point and 1000 time-point images), for three subjects. MRF-SOHO shows similar quality to zero-filled MRF despite a relative acceleration factor of ~4.6x. T_1_ and T_2_ values in white matter, grey matter and cerebrospinal fluid (CSF) obtained with zero-filled MRF and the accelerated MRF-SOHO are shown in Table 1, for all subjects. Zero-filled MRF generally underestimated T_1_ in white matter and generally overestimated T_2_ in grey matter when compared with literature values [30, 31, 32, 33]. This bias was not observed with the proposed MRF-SOHO despite the scan acceleration. Considerable variations in T_1_ and T_2_ in CSF were observed with both methods in different subjects. Challenges in mapping CSF due to high parametric values, flow and other errors have been reported in other works [34, 35] and require further study.

**Table 1:** T_1_ and T_2_ in healthy subjects.

	T_1_ zero-filled	T_1_ SOHO	Literature T_1_	T_2_ zero-filled	T_2_ SOHO	Literature T_2_
Subject 1: white matter	504±98	691±84	608–756	67±10	52±10	54–81
Subject 1: grey matter	976±152	1146±149	998–1304	118±27	89±17	78–98
Subject 1: CSF	3913±176	3917±220	4103–5400	1941±170	2576±66	1800–2460
Subject 2: white matter	497±53	649±51	608–756	65±11	57±10	54–81
Subject 2: grey matter	959±100	1058±87	998–1304	100±22	90±21	78–98
Subject 2: CSF	3859±148	3821±187	4103–5400	1800±207	2362±336	1800–2460
Subject 3: white matter	627±48	674±54	608–756	54±4	52±6	54–81
Subject 3: grey matter	1064±120	1121±122	998–1304	83±14	80±13	78–98
Subject 3: CSF	2971±407	3057±359	4103–5400	777±115	653±138	1800–2460

**Fig 7:**
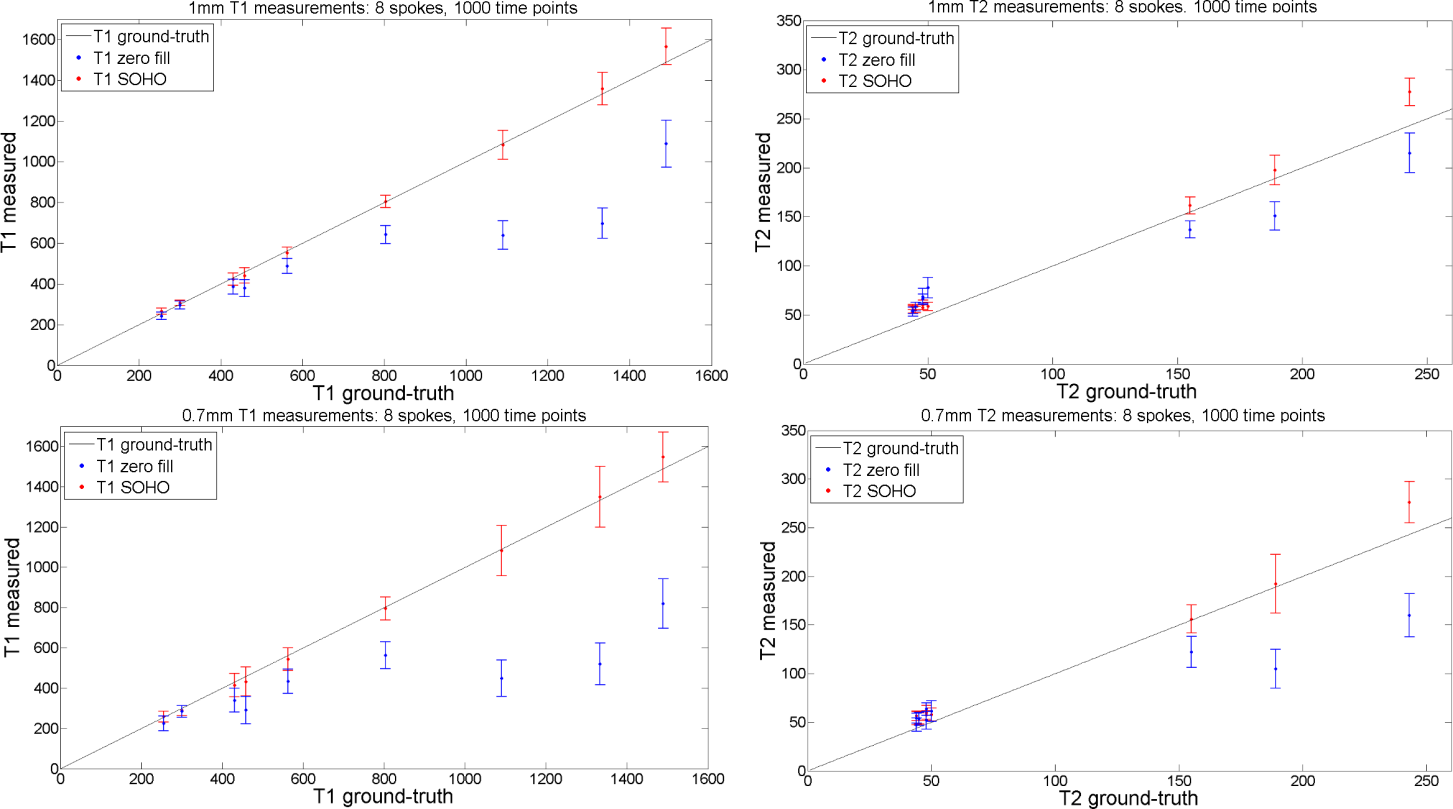
T_1_ and T_2_ measurement plots (in ms) with zero-filled MRF and the proposed MRF-SOHO reconstructions, using 8 spokes, 1000 time points for 1mm resolution and 0.7 mm^2^ resolution. Analogous to the results in Fig 3, zero-filled MRF underestimated T_1_, particularly at higher resolutions. A slight overestimation of T_2_ was observed for the proposed approach, whereas zero-filled MRF considerably underestimated T_2_ due to the higher undersampling and reduced SNR.

Plots of measured T_1_ and T_2_ values in the phantom with 1x1 mm^2^ and 0.7x0.7 mm^2^ datasets using 8 radial profiles per time-point and 1000 time-point images are shown in Fig 7. The increased undersampling factors per time-point image (~51x for 1x1mm^2^ and ~73x for 0.7x0.7mm^2^ with 8 radial profiles) and reduced signal to noise ratios (SNR) from higher resolutions introduce some errors in the parametric maps using zero-filled MRF, whereas superior accuracy and precision is obtained with the proposed MRF-SOHO.

Parameter maps for the standardized phantom using 8 spokes and 1000 time points reconstructed with zero fill and SOHO are shown in S1 Fig, in correspondence to the plots in Figs 3 and 7. Underestimation bias is observed with zero-filled MRF at higher resolution data (due to higher undersampling factors), whereas accuracy and precision is generally maintained with SOHO. A loss in apparent SNR is observed in both methods, however this degradation is also reduced with SOHO.

**Fig 8:**
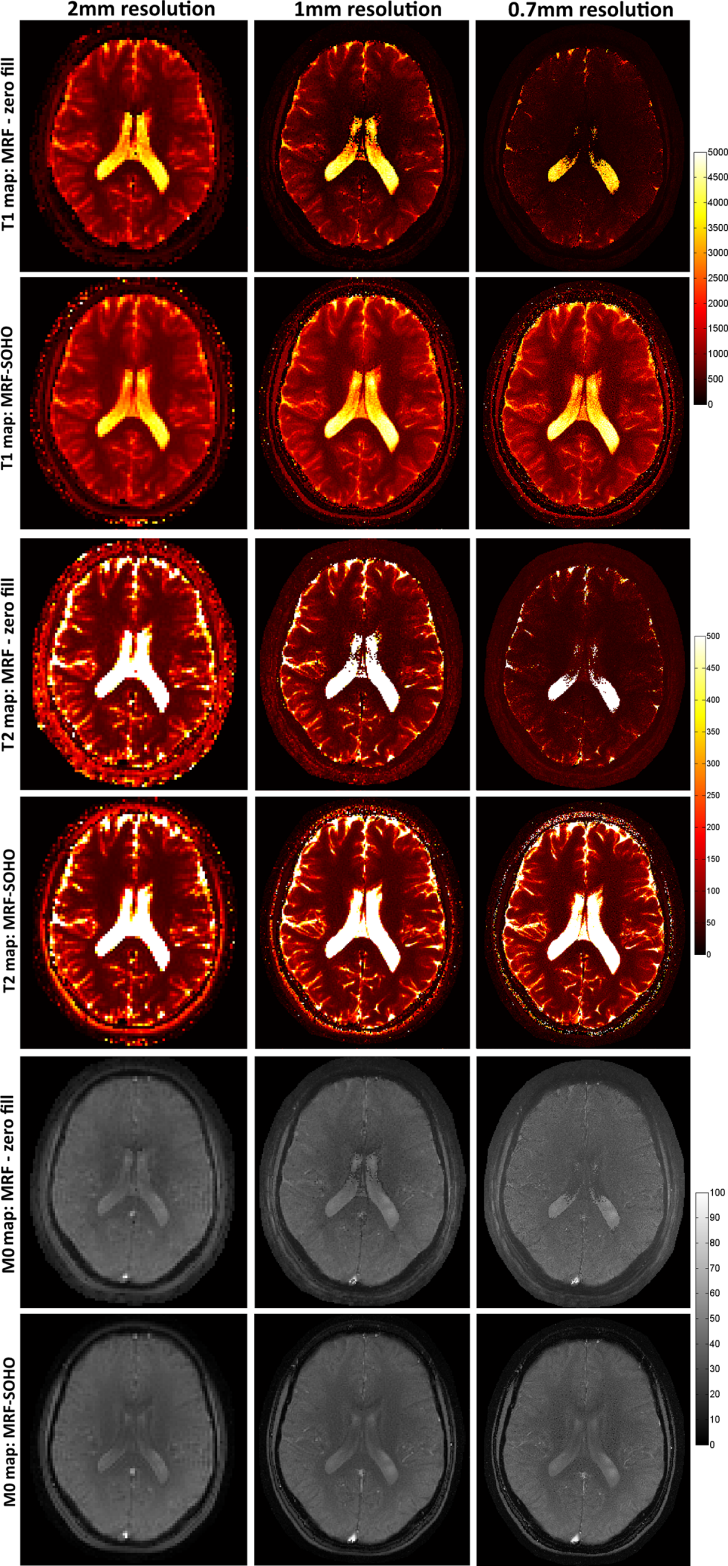
T_1_, T_2_ (in ms) and M_0_ maps for zero-filled MRF reconstruction and MRF-SOHO using 8 spokes, 1000 time points for 2 mm^2^, 1 mm^2^ and 0.7 mm^2^ resolution in subject 1. Both methods produce comparable parametric maps at 2 mm^2^ resolution, however zero-filled MRF quickly degrades at higher resolutions (and implicitly higher undersampling and noise factors). MRF-SOHO maintained parametric map quality, however a loss in SNR was also observed.

T_1_, T_2_ and M_0_ parametric maps for subject 1 using 2x2 mm^2^, 1x1 mm^2^ and 0.7x0.7 mm^2^ datasets are shown in Fig 8 for zero-filled MRF and the proposed MRF-SOHO using 8 radial profiles and 1000 time points. The proposed MRF-SOHO outperforms zero-filled MRF across all resolution datasets. This is particularly evident at higher resolutions (0.7x0.7 mm^2^), where zero-filled MRF shows considerable degradation due to lower SNR and higher undersampling factor. MRF-SOHO was robust to these conditions, however a reduction in SNR was observed at higher accelerations. The ratio of mean by standard deviation in a ROI in T_1_ and T_2_ maps was taken as a surrogate for parametric map SNR. Estimated SNR in T_1_ for zero-filled reconstruction was approximately 10.7, 4.3 and 4.5, for resolutions 2x2 mm^2^, 1x1 mm^2^ and 0.7x0.7 mm^2^, respectively; corresponding results for SOHO were 26.0, 9.2 and 4.8. Estimated SNR in T_2_ for zero-filled reconstruction was approximately 11.6, 5.6 and 5.5, for resolutions 2x2 mm^2^, 1x1 mm^2^ and 0.7x0.7 mm^2^, respectively; corresponding results for SOHO were 18.2, 9.6 and 5.2. Considerable higher SNR was generally observed for SOHO when compared with a zero-filled reconstruction. At higher resolutions, the SNR estimates in zero-filled MRF were affected by the substantial underestimation bias observed in the parametric maps (Fig 8). A similar analysis (not shown here) to the one performed for 2x2mm^2^ resolution dataset suggests the use of 5 profiles, 700 time point images for 1x1 mm^2^ resolution and 8 radial profiles, 700 time-point images for 0.7x0.7 mm^2^ resolution as the most adequate accelerations for MRF-SOHO, respectively. The datasets with 2x2mm^2^, 1x1mm^2^ and 0.7x0.7mm^2^ were acquired in approximately 29, 42 and 52 seconds, respectively. Retrospectively accelerated by SOHO, these scan times were reduced to approximately 6, 18 and 23 seconds.

## Discussion

A novel approach for accelerated magnetic resonance fingerprinting acquisition was introduced and validated in a standardized phantom and healthy subject data. The proposed MRF-SOHO combines parallel imaging, soft-weighting and key-hole concepts to reduce aliasing in the highly undersampling time-point images and improves resulting parametric maps. The method shares primarily high frequency information between consecutive time-point images, which is redundant since image contrast evolves slowly when the flip angle is varied smoothly. MRF-SOHO enabled an acceleration factor of ~4.6x relative to zero-filled MRF (considering 8 radial profiles per time-point image and 1000 time points). It was also shown that MRF-SOHO enables sub-millimetric MRF, whereas zero-filled MRF was not robust due to highly increased undersampling artefacts of the time-point images and decreased voxel signal. Additionally, the proposed approach produces high quality time-point images, which may have high diagnostic value when used as a complement to the resulting parametric maps. Alternatively, the unalised time-points could be used for motion estimation, enabling motion correction for MRF [36].

T_1_ and T_2_ values measured with the proposed approach were generally in agreement with ground-truth phantom measurements and literature values for brain tissues. However, T_2_ overestimation was observed in phantom measurements. T_2_ overestimation has been observed before in [34, 35] and can be addressed with optimised patterns for FA and TR. T_2_ bias was also observed for both methods in the CSF, which has also been reported before in [8, 22]. The simulated dictionary B_1_ was fixed at 1.0, however B_1_ inhomogeneity may introduce errors in the estimated maps, among them the T_2_ bias observed for the CSF. Slice profile imperfections in addition to B_1_ inhomogeneities have also shown to produce bias in MRF [37]. Flow in the CSF and partial volume effects may also lead to errors in CSF measurements. Additionally, magnetization transfer [38] and coil receiver bias [39] have also been shown to affect T_1_, T_2_ and M_0_ estimation with MR fingerprinting. Zero-filled MRF presented less accurate phantom results, increased errors at sub-millimetric resolution and/or high acceleration and underestimation of T_1_ in the range of white matter and overestimation of T_2_ in grey matter. MRF-SOHO produced more accurate parametric maps, despite the relative acceleration factor of 4.6x.

In this work we use a model-based approach for a soft-weighted key-hole reconstruction and determine the data sharing parameters (*α*, *β*, *μ*, *τ*, *N*) experimentally, assuming a slow varying flip angle. Incorrect choice of these parameters could affect image contrast of the time-points and potentially introduce a bias in the resulting parameter maps. The optimal data sharing parameters will depend primarily in the sequence parameters (e.g. flip angle pattern) and parameters will have to be tuned for each MRF sequence. However, a data-based approach may provide a more general solution. For instance, singular value decomposition may be used to compress the dictionary and acquired data into a space with reduced number of time points [40]. This analysis is considered as future work and it may reveal further redundancies in the data and therefore enable higher acceleration factors. As MRF will generally operate in highly undersampled regimes, aliasing artefacts may propagate into the parametric maps. Methods like soft-weighting, sliding window, parallel imaging and compressed sensing can reduce these errors. Future work should evaluate the improvements of these different strategies, separately and combined, to investigate how these approaches may be used to accelerate MRF. In this work a golden radial trajectory is used instead of the commonly used spiral trajectory for MRF, to avoid T_2_ decay induced blurring and avoid acquisition of repeated k-space positions. However, the proposed reconstruction method is applicable to any trajectory, as long as enough low frequency information is acquired for every time-point and k-space positions within SOHO’s shared neighbourhood are minimized to maximize trajectory efficiency.

As in [7], a single inversion pulse is used in the beginning of the acquisition to improve sensitivity to T_1_. The results in [14] demonstrate the improvement of additional magnetization preparation pulses in MRF parametric mapping. Future work should explore additional preparation pulses and combine them with the proposed MRF-SOHO framework. Additionally, improved sensitivity to T_1_ and T_2_ (and improved SNR) could be achieved with a pseudo b-SSFP [41]. Recent work [35] has also demonstrated the benefit of optimized FA and TR patterns to improve estimation of T_1_ and especially T_2_. Future work should also incorporate B_1_ into the MRF model, as it has been proposed in [12, 13, 37, 38]. Another main limitation of the proposed approach is the reconstruction time (up to 120 minutes for 0.7x0.7 mm). Currently, the method was implemented in MATLAB, however this can be significantly reduced with GPU implementations [42]. In this study, data was retrospectively undersampled to allow comparisons of different acceleration factors from the same dataset. When evaluating the number of time-points, a subset of the full set of reconstructed images were retrospectively taken, meaning that the final time-points had access to slightly more high frequency information than in a prospective acquisition, however we consider this difference to be small. The number of golden radial profiles was also retrospectively studied. This means that reconstructions with 5 and 2 profiles do not have a golden angle separation between all angles and may provide suboptimal image quality. A prospective acquisition with 5 radial profiles will potentially have less residual artefacts than the corresponding retrospective acquisition, improving MRF mapping quality and potentially enabling even higher acceleration factors. This work focused on demonstrating the feasibility of MRF-SOHO in healthy subjects. Future work will evaluate this approach in patient studies.

## Conclusion

A novel approach for accelerated magnetic resonance fingerprinting (MRF-SOHO) was proposed, achieving comparable parametric maps to zero-filled MRF despite a relative acceleration factor of 4.6x. When using the same acceleration factor, the proposed approach produced superior parametric maps in sub-millimetric MRF when compared to zero-filled MRF.

## Supporting information

S1 Video First 500 time-points for zero-filled gridding (**left**) and MRF-SOHO (**right**) for a 2 mm^2^ resolution dataset.
(AVI)

S2 Video First 500 time-points for zero-filled gridding (**left**) and MRF-SOHO (**right**) for a 1 mm^2^ resolution dataset.
(AVI)

S3 Video First 500 time-points for zero-filled gridding (**left**) and MRF-SOHO (**right**) for a 0.7 mm^2^ resolution dataset.
(AVI)

S1 Fig Parametric maps for the standardized phantom using 1000 time-points and 8 spokes, reconstructed with zero-filled MRF and the proposed SOHO. Underestimation bias is observed with zero-filled MRF at higher resolution data (due to higher undersampling factors), whereas accuracy and precision is generally maintained with SOHO. A loss in apparent SNR is observed in both methods, however this degradation is also reduced with SOHO.
(TIF)
