# Supplementary figures and images for: Accelerated magnetic resonance fingerprinting using soft-weighted key-hole (MRF-SOHO)
Source: PLoS One. 2018 Aug 9;13(8):e0201808. doi: 10.1371/journal.pone.0201808 (PMC6084944; doi:10.1371/journal.pone.0201808)

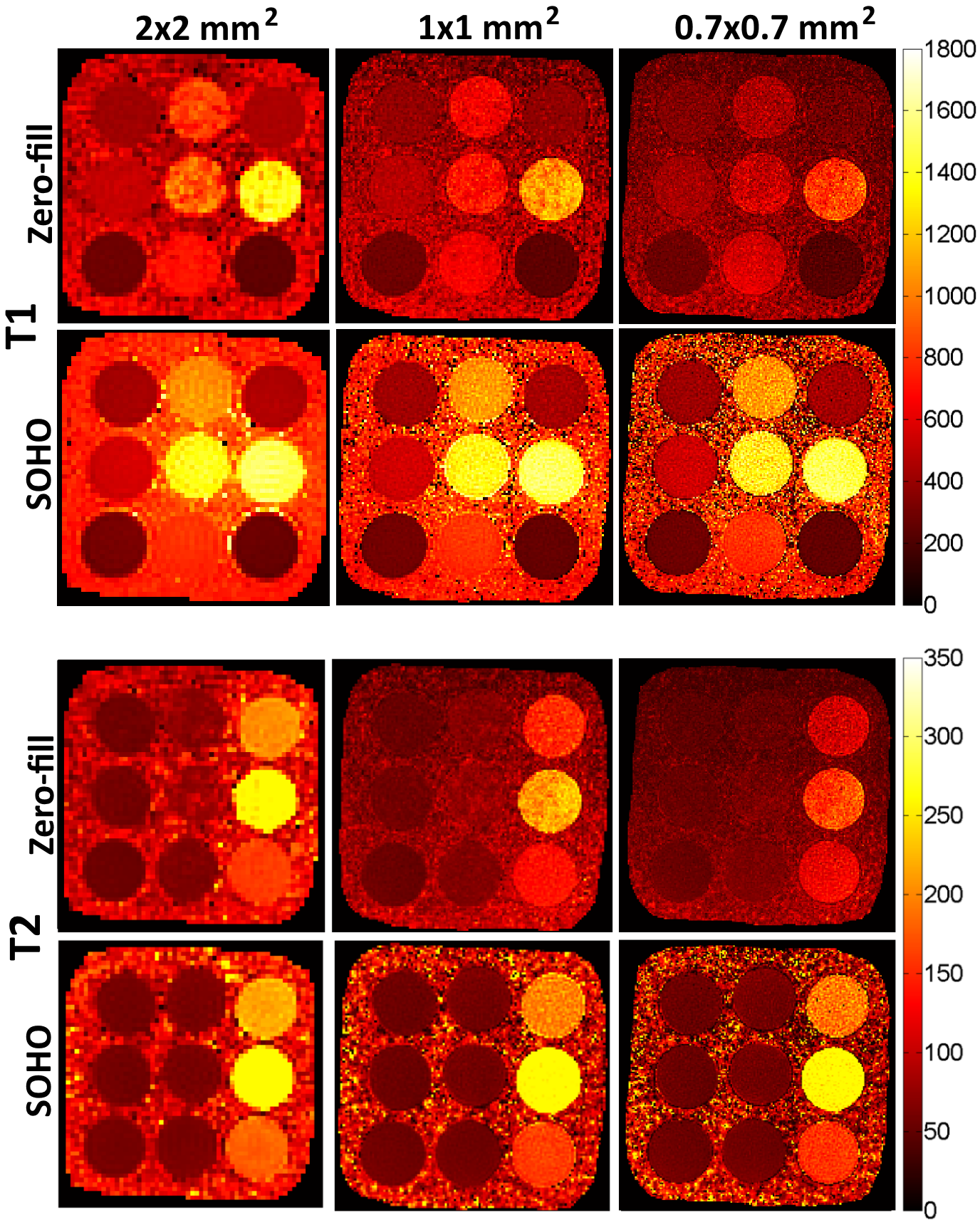

Supplement: S1 Fig — Underestimation bias is observed with zero-filled MRF at higher resolution data (due to higher undersampling factors), whereas accuracy and precision is generally maintained with SOHO. A loss in apparent SNR is observed in both methods, however this degradation is also reduced with SOHO. (TIF) [file pone.0201808.s004.tif]
